# Limb salvage and survival after urgent surgical treatment of popliteal artery aneurysm

**DOI:** 10.1186/s13017-023-00514-7

**Published:** 2023-10-14

**Authors:** Sara Pomatto, Gianluca Faggioli, Rodolfo Pini, Ilaria Ficarelli, Alessia Pini, Cecilia Angherà, Cristina Rocchi, Stefania Caputo, Andrea Vacirca, Carlo Ruotolo, Mauro Gargiulo

**Affiliations:** 1 Vascular Surgery, Department of Medical and Surgical Sciences (DIMEC), University of Bologna “Alma Mater Studiorum” - DIMEC, Policlinico S. Orsola, Via Giuseppe Massarenti 9, 40138 Bologna, Italy; 2 Vascular Surgery Unit, IRCCS Azienda Ospedaliero-Universitaria S. Orsola, Bologna, Italy; 3 Division of Vascular Surgery, Cardarelli Hospital, 9 Via A. Cardarelli, 80131 Naples, Italy

**Keywords:** Popliteal artery aneurysm, Urgent, Surgical treatment, Thrombolysis, Acute limb ischemia, Rupture, Limb salvage, Survival

## Abstract

**Background:**

Popliteal artery aneurysms (PAAs) need urgent treatment in case of acute thrombosis, distal embolization, or rupture. Few data are available in the literature about the treatment results in these scenarios. The aim of the present study was to evaluate an 11-year multicenter experience in the urgent treatment of PAAs.

**Methods:**

All symptomatic PAAs surgically treated in two vascular centers between 2010 and 2021 were retrospectively analyzed. In the postoperative period periodical clinical and Duplex-Ultrasound evaluation were performed. The evaluated endpoint was the outcome of urgent PAAs treatment according to their clinical presentation. Statistical analysis was performed by Kaplan-Meier log-rank evaluation and multivariable Cox regression tests.

**Results:**

Sixty-six PAAs needed an urgent repair. Twelve (18%) patients had a PAA rupture and 54 (82%) had an acute limb ischemia (ALI) due to either distal embolization or acute thrombosis. Patients with ALI underwent bypass surgery in 51 (95%) cases, which was associated with preoperative thrombolysis in 18 (31%) cases. A primary major amputation was performed in 3 (5%) cases. The mean follow-up was 52 ± 21 months with an overall 5-year limb salvage of 83 ± 6%. Limb salvage was influenced only by the number of patent tibial arteries (pTA) [5-years limb salvage 0%, 86 ± 10%, 92 ± 8% and 100% in case of 0, 1, 2 or 3 pTA, respectively (*P* = .001)]. An independent association of number of pTA and limb loss was found [hazard ratio (HR): 0.14 (95% confidence interval (CI) 0.03–0.6), *P* = .001]. Overall 5-year survival was 71 ± 7%. Ruptured PAAs were associated with lower 5-year survival compared with the ALI group (48 ± 2% vs. 79 ± 7%, *P* = .001). The number of pTA (33 ± 20%, 65 ± 10%, 84 ± 10% and 80 ± 10% for 0, 1, 2 and 3 pTA, respectively, *P* = .001) and the thrombolysis (94 ± 6% vs. 62 ± 10%, *P* = .03) were associated with higher survival in patients with ALI. There was an independent association of number of pTA and long-term survival [HR 0.15 (95% CI 0.03–0.8), *P* = .03].

**Conclusions:**

PAA rupture is the cause of urgent PAA treatment in almost one fifth of cases, and it is associated with lower long-term survival. ALI can benefit from thrombolysis, and long-term limb salvage and survival are associated with the number of pTA.

## Background

Popliteal artery aneurysms (PAAs) represent 70% of all peripheral arterial aneurysms [1]. PAAs are mostly asymptomatic and incidentally detected. Fourteen to 24% of the asymptomatic patients will become symptomatic within 1–2 years and 31–68% will develop complications during their lifetime [2–6]. PAAs need urgent treatment in case of acute limb ischemia (ALI) due to acute aneurysm thrombosis or distal embolization to tibio-pedal arteries and in the cases of aneurysm rupture [6].

In patients with ALI, intervention should be defined according to the severity of ischemia (Rutherford clinical categories of ALI) [7]. In case of PAA thrombosis and loss of tibio-pedal runoff arteries, with mild/moderate ALI, intra-arterial thrombolysis is indicated to restore runoff for a subsequent surgical treatment. Patients with limb threatening ischemia should urgently undergo surgical or endovascular revascularization, possibly with adjunctive procedures as mechanical or aspiration thrombectomy of runoff vessels. In case of irreversible ALI, a primary major amputation is needed [6].

Ruptured PAA can threaten both patient’s limb and life and can be surgically treated with ligation or bypass grafting. Endovascular treatment utilizing stent-graft placement is also a viable option.

Despite their clinical relevance, only few data are available in the literature about the results of PAAs urgent surgical treatment in these different scenarios. The aim of the present study was to evaluate an 11-year experience in the urgent treatment of PAAs according to their clinical presentation (ALI or rupture) in two vascular centers.

## Methods

### Patients selection

All patients surgically treated for symptomatic PAA in two vascular centers (Vascular Surgery Unit, IRCCS University Hospital Policlinico Sant’Orsola, Bologna, and Vascular Surgery Unit, Cardarelli Hospital, Napoli) from January 2010 to December 2021 were prospectively collected into a dedicated database and retrospectively analyzed. The inclusion criteria were the presence of a symptomatic PAA with acute limb ischemia due to acute thrombosis or distal embolization or with symptoms related to PAA rupture.

### Patients characteristics

Demographic and clinical characteristics of the enrolled patients included the following: sex, age, hypertension (defined as systolic blood pressure ≥ 140 mmHg or diastolic blood pressure ≥ 90 mmHg), dyslipidaemia (defined as total cholesterol level ≥ 200 mg/dL or low density lipoprotein level ≥ 120 mg/dl or specific therapy), diabetes mellitus (pre-diagnosed in therapy with oral hypoglycaemic drugs or insulin), coronary artery disease (CAD, defined as history of angina pectoris, myocardial infarction or coronary revascularization), chronic obstructive pulmonary disease (COPD, defined as chronic bronchitis or emphysema), active smoking, chronic kidney disease (CKD, defined as glomerular filtration rate < 30 ml/min), obesity (defined as a Body Mass Index ≥ 30) and atrial fibrillation. Data about concomitant contralateral PAA and aorto-iliac aneurysm were collected.

Clinical presentation (ischemia or rupture related symptoms and grade of ALI according to Rutherford classification [7]), anatomical features of the aneurysm (diameter, thrombotic involvement, extension) and the number of patent tibial arteries (pTA) were preoperatively evaluated. Thrombolytic intra-arterial therapy was administrated in case of PAA thrombosis/embolization and loss of tibio-pedal runoff arteries, with mild/moderate ALI in the absence of absolute and relative major and/or minor contraindications prior to its initiation. [9]

### Diagnosis and preoperative assessment

After physical examination, all patients underwent an urgent lower limbs Computed Tomography Angiography (CTA) to assess the diameter and the extension of the aneurysm and the amount of PAA thrombosis or the presence of rupture signs. The examination was usually extended to aorto-iliac arteries to detect the presence of concomitant aneurysms.

In case of PAA acute thrombosis or distal embolization, a lower limb angiography was performed to evaluate the tibio-pedal arteries run-off. If no outflow vessel was identified at preoperative angiography and the patient’s limb was not immediately threatened (Rutherford IIa ALI [7]), catheter-directed intra-arterial thrombolytic therapy (Urokinase) was delivered to restore blood flow to potential outflow target arteries. Urokinase was administered as a bolus of 100,000 I.U., followed by continuous infusion of 50,000–70,000 I.U./24 h. Concomitant intravenous continuous sodic heparin infusion was associated to maintain an aPTT value two times higher than baseline values. Angiographic evaluations were performed at least every 24 h and thrombolysis was carried out for a maximum of 3 days.

The great saphenous vein (GSV) was also evaluated preoperatively by Duplex Ultrasound (DUS) and was considered suitable if > 3 mm in diameter and without significant wall thickening or intraluminal thrombosis.

### Surgical procedure

The surgical technique was chosen according to the PAA extension, the number of patent tibio-pedal arteries and the GSV suitability. In case of an inadequate GSV an alternative bypass material (ePTFE graft) was employed.

A medial or posterior approach was chosen according to the anatomical features of the aneurysm (extension, involvement of superficial femoral artery) and the surgeon preference.

Surgery was performed under general or spinal anesthesia. All patients underwent broad spectrum antibiotic infusion and systemic heparinization (60–80 IU/kg).

Patients with irreversible limb ischemia with motor and sensory loss of function at presentation (Rutherford III [7]) underwent primary major amputation.

### Perioperative and long-term outcomes

Bypass patency and tibio-pedal runoff were assessed for each patient through clinical and DUS examinations before the discharge. Postoperative mortality was considered at 30 days from the intervention.

In the postoperative period clinical and DUS examination were performed at 1, 3, 6, 12 months and yearly thereafter to evaluate survival, graft patency and limb salvage.

### Statistical analysis

All categorical variables were expressed as frequencies and compared using Fisher’s exact test; continuous variables were expressed with median and interquartile range (IQR) and compared using Mann Withey U. Moreover, late survival and limb salvage rates were evaluated using Kaplan-Maier and compared using log-rank test. Cox proportional hazards model, expressed with hazard ratio (HR) and 95% confidence interval (CI), was used to identify predictors for major amputation.

In all the statistical tests *p* values (2-tails) of 0.05 or less were considered statistically significant. The statistical analysis was performed using SPSS 23.0 for Apple (SPSS Inc, Chicago, Illinois, USA).

## Results

### Clinical presentation and perioperative results

**Fig. 1:**
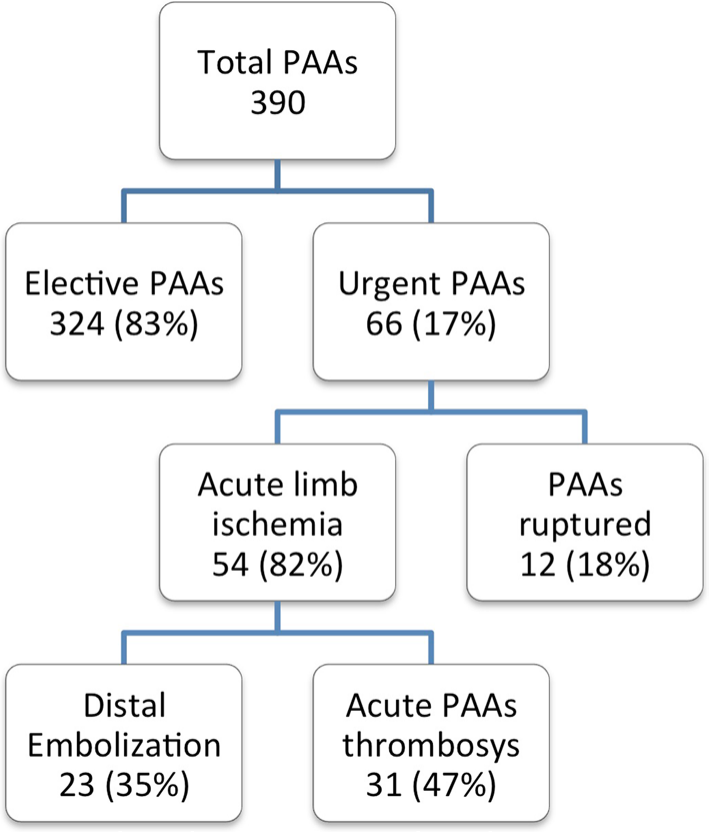
Population with popliteal artery aneurysm treated during the study period. *PAA* popliteal artery aneurysm

In the examined period a total of 390 PAAs were surgically treated in two vascular centers; 66 (17%) of them needed an urgent treatment and were included in the present study, Fig. 1.

Median age of the patients was 72 (IQR: 18) years and they were all males (100%). Preoperative demographic and clinical characteristics are listed in Table 1.

**Table 1:** Preoperative demographic and clinical characteristics of the evaluated patients

	All urgent PAA (66 pts) % (n)	PAA rupture (12 pts) % (n)	PAA acute ischemia (54 pts) % (n)	*P*
Male	100% (66)	100% (12)	100% (54)	1.0
**Age**^**a**^** (years)**	72 (18)	86 (16)	69 (17)	**.001**
Hypertension	79% (52)	92% (11)	79% (41)	1.0
Dyslipidaemia	16% (24)	52% (6)	33% (18)	.34
**Diabetes mellitus**	12% (8)	33% (4)	8% (4)	**.03**
Coronary artery disease	5% (7)	12% (2)	8% (5)	1.0
Chronic obstructive pulmonary disease	30% (20)	42% (5)	28% (15)	.31
Smoke	44% (29)	25% (3)	48% (26)	.45
**Chronic kidney disease**	11% (7)	33% (4)	6% (3)	**.03**
Obesity	7% (5)	8% (1)	7% (4)	1.0
Atrial fibrillation	14% (9)	33% (4)	9% (5)	.18
PAA diameter mm^a^	39 (19)	25 (45)	37 (18)	.30

Statistically significant values are given in bold

*PAA* popliteal artery aneurysm

^a^Median and interquartile range

Median PAA diameter was 39 mm (IQR: 19 mm). Twelve (18%) patients had a PAA rupture and 54 (82%) had an acute limb ischemia due to either distal embolization (23; 35%) or acute PAA thrombosis (31; 47%). Among patients with ALI, 28 (52%) had moderate (Rutherford IIa [7]), 23 (43%) limb threatening (Rutherford IIb [7]) and 3 (5%) irreversible (Rutherford III [7]) ALI, respectively.

Patients with PAA rupture were older than those presenting with ischemic symptoms (82 ± 9 vs 69 ± 10 year, *P* = 0.01) and they were treated with a prosthetic graft in most cases (11/12; 92%).

Patients with acute limb ischemia (Rutherford IIa and IIb [7]) were treated with bypass surgery in 51 (94%) cases. Preoperative intra-arterial thrombolysis was administrated in 18 (33%) cases and intraoperative distal thrombectomy in 12 (22%). Revascularization was performed using an autologous great saphenous vein graft in 20 (37%) cases. In 10 (19%) patients fasciotomies were performed after revascularization to avoid compartment syndrome.

A primary major amputation was performed in 3 (5%) cases because of the presence of irreversible ischemia (Rutherford III [7]) at presentation (Table 2).

**Table 2:** Surgical technical aspects

	All urgent PAA (66)	PAA rupture (12)	PAA acute ischemia (54)	*P*
Preoperative intra-arterial fibrinolysis	18 (27%)	0 (0%)	18 (33%)	–
Vein graft	21 (32%)	1 (8%)	20 (37%)	–
Prosthetic graft	45 (68%)	11 (92%)	31 (57%)	.06
Posterior approach	1 (2%)	0 (0%)	1 (2%)	.32
Tibial artery intra-operative thrombectomy	13 (20%)	1 (8%)	12 (22%)	.32
Fasciotomy	10 (15%)	0 (0%)	10 (19%)	.21
Primary major amputation	3 (4%)	0 (0%)	3 (5%)	.50

Only one death occurred in the perioperative period (30-day mortality 2%) in a patient with a ruptured PAA.

### Long-term outcomes

**Fig. 2:**
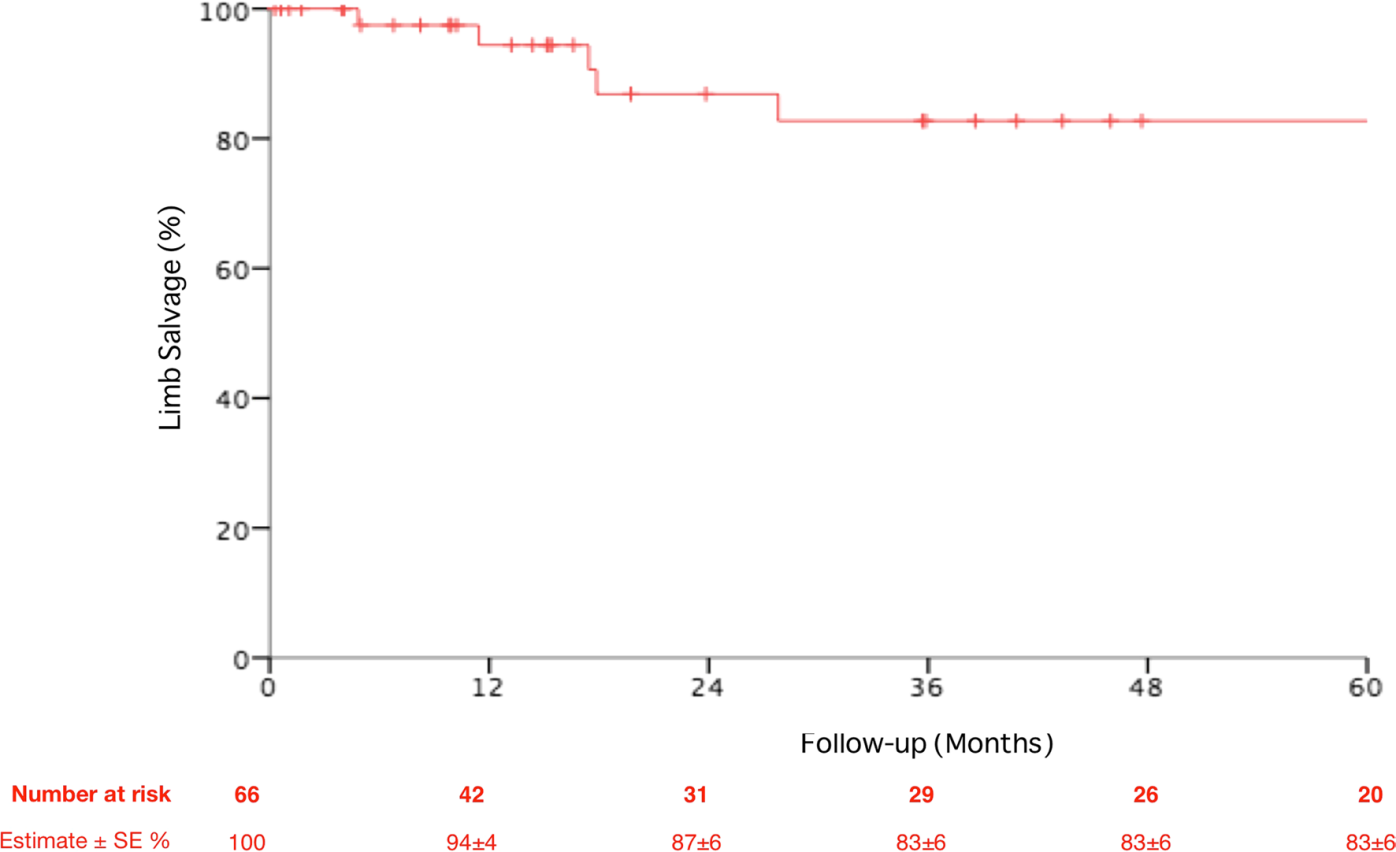
Overall limb salvage of the population of the study during the follow-up period. *SE* standard error

The mean follow-up was 52 ± 21 months with an overall 5-year limb salvage of 83 ± 6%, Fig. 2.

**Fig. 3:**
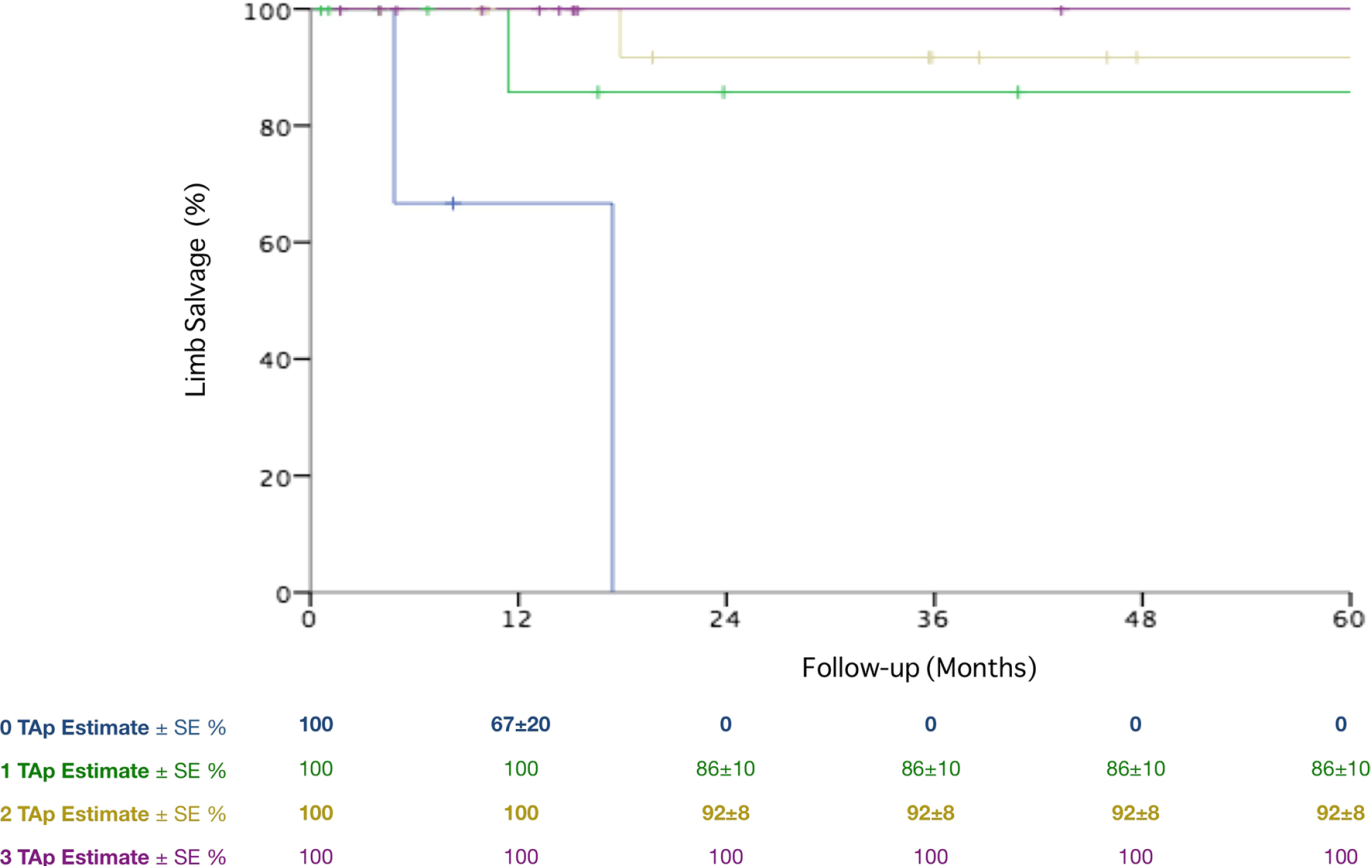
Long-term limb salvage according to the number of patent tibial arteries. *pTA* patent tibial arteries, *SE* standard error

Limb salvage during the follow-up period was influenced only by the number of patent tibial arteries (pTA): at 5 years 0%, 86 ± 10%, 92 ± 8% and 100% in case of 0, 1, 2 or 3 pTA, respectively (*P* = 0.001) (Fig. 3).

Clinical presentation and bypass graft material were not associated with limb loss during the follow-up period. Cox regression confirmed the independent association of number of pTA and limb loss during the follow-up: HR 0.14 and 95% CI 0.03–0.6, *P* = 0.001.

**Fig. 4:**
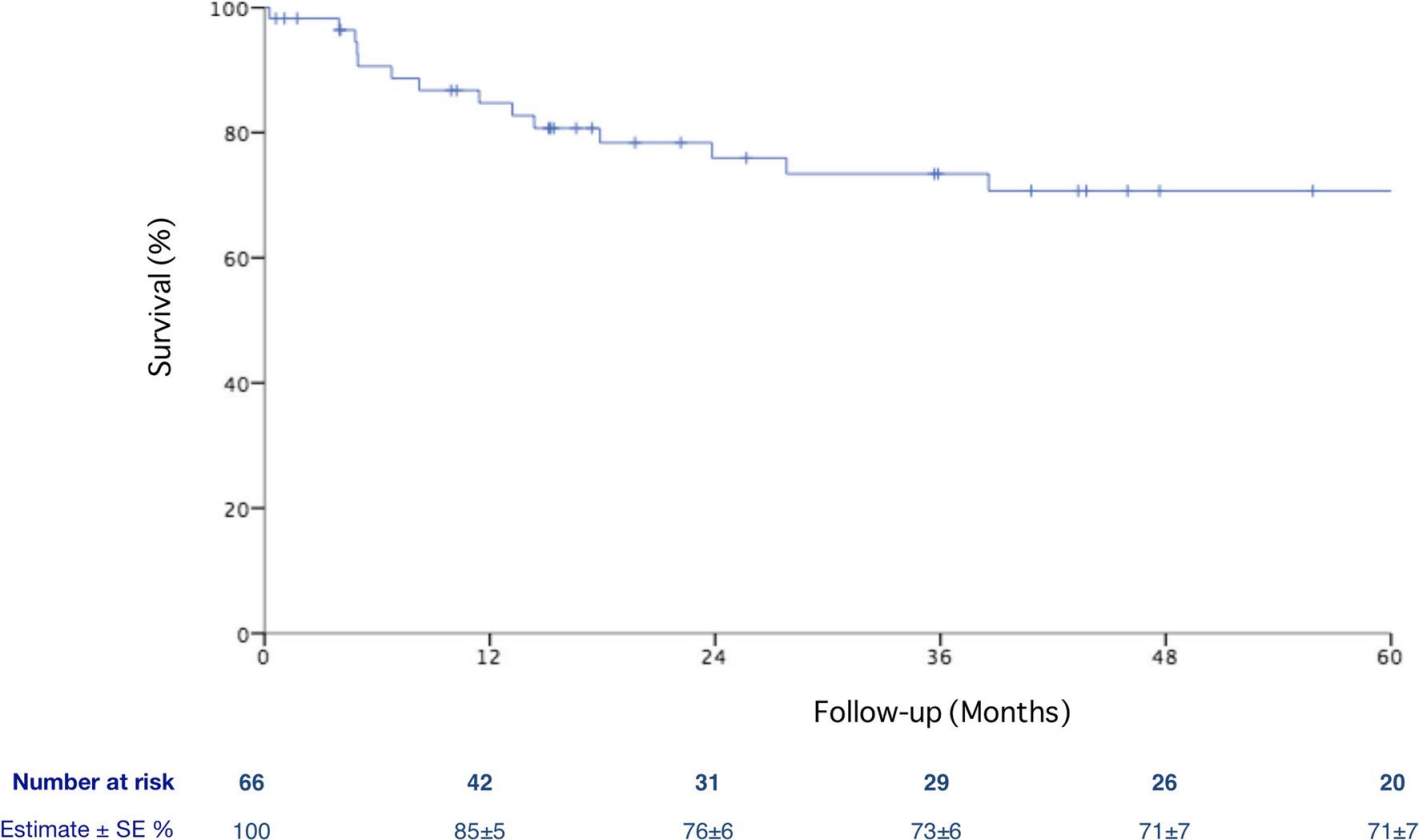
Overall survival of the population of the study during the follow-up period. *SE* standard error

**Fig. 5:**
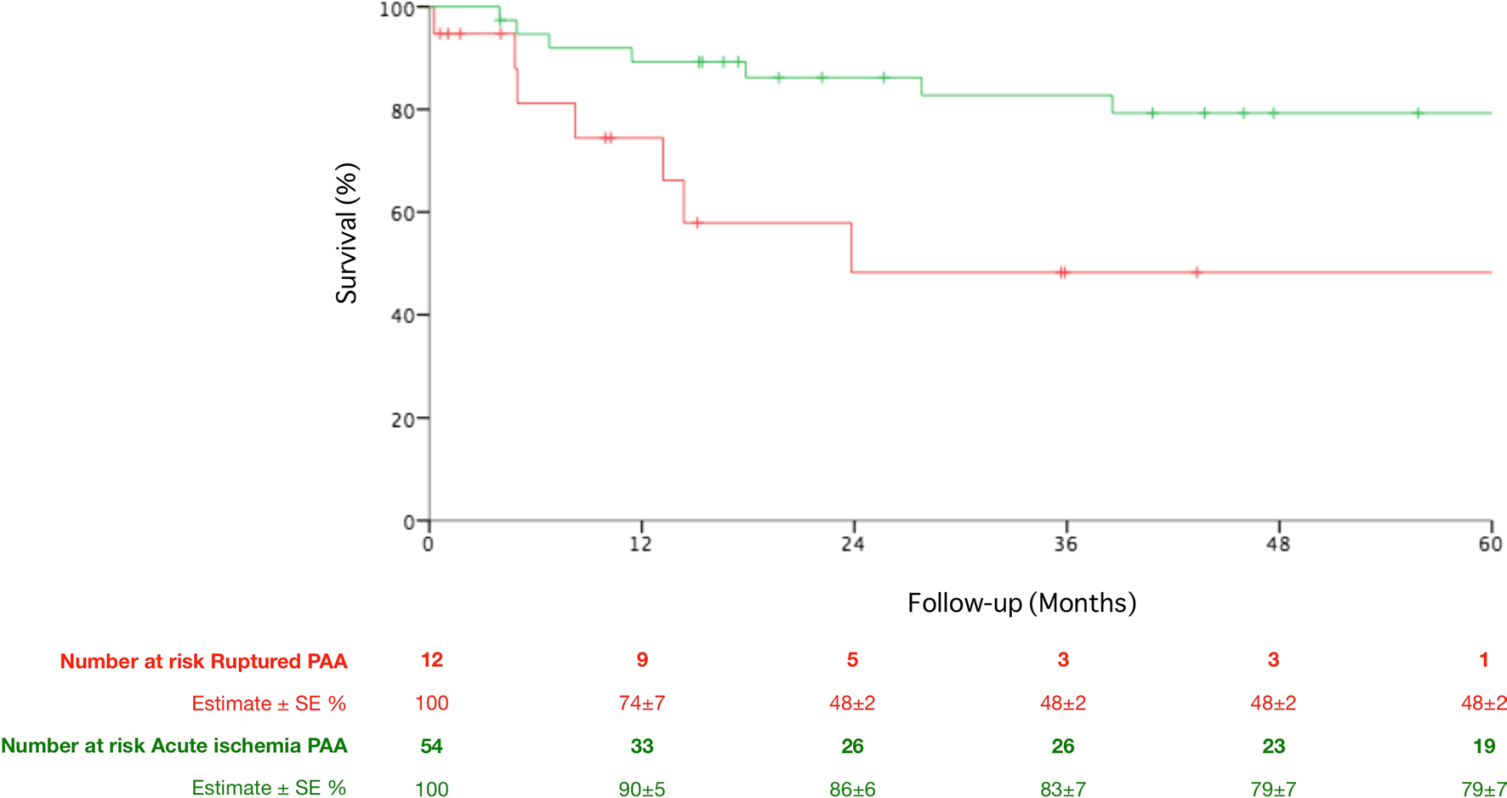
Long-term survival of urgent popliteal artery aneurysms treated for rupture and acute limb ischemia. *PAA* popliteal artery aneurysm, *SE* standard error

**Fig. 6:**
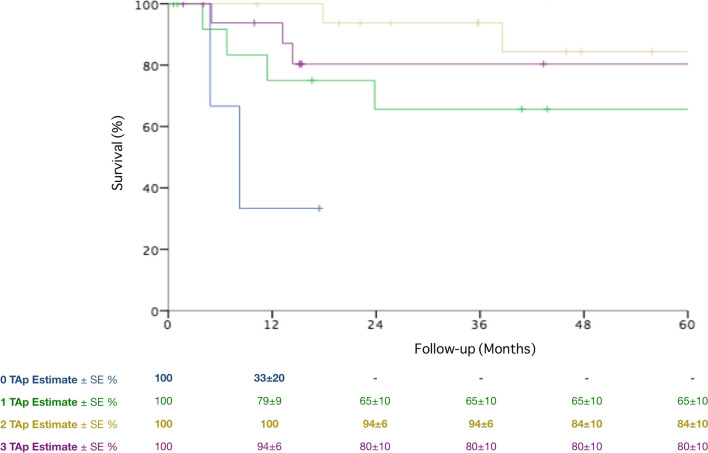
Long-term survival according to the number of patent tibial arteries in patients with acute limb ischemia at presentation. *pTA* patent tibial arteries, *SE* standard error

**Fig. 7:**
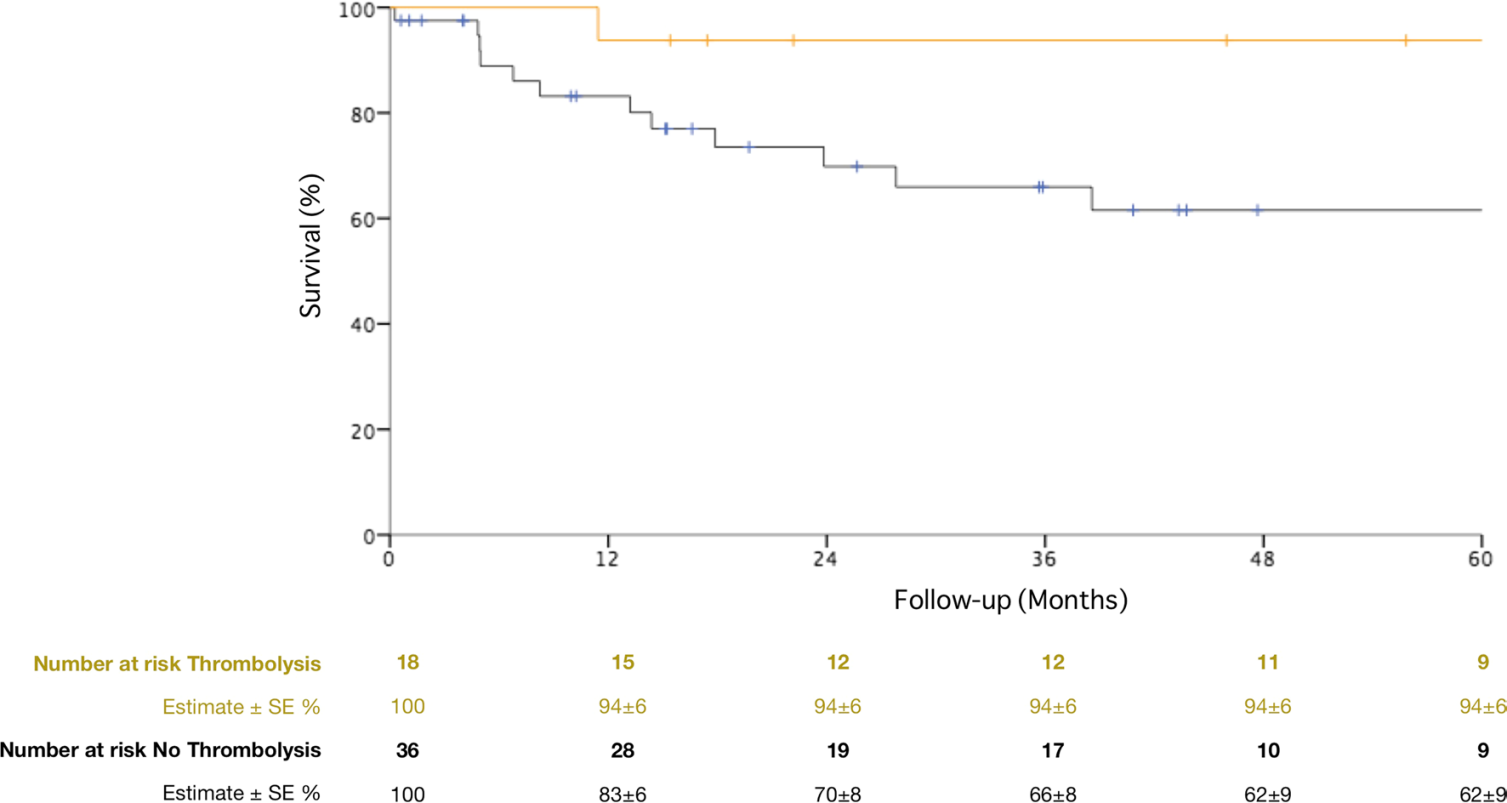
Long-term survival in patients undergoing preoperative intra-arterial thrombolytic therapy versus no thrombolysis group in patients with acute limb ischemia at presentation. *SE* standard error

Overall, 5-year survival after urgent PAA treatment was 71 ± 7%, Fig. 4. Ruptured PAAs were associated with a lower 5-year survival rate compared with patients treated for ALI (48 ± 2% vs. 79 ± 7%, *P* = 0.001, Fig. 5). Moreover, patients with ALI at presentation had a higher survival according to the number of pTA (33 ± 20%, 65 ± 10%, 84 ± 10% and 80 ± 10% for 0, 1, 2 and 3 pTA, respectively, *P* = 0.001, Fig. 6) and the preoperative thrombolysis administration (94 ± 6% vs. 72 ± 10%, *P* = 0.03, Fig. 7). Cox regression confirmed the independent association of number of pTA and survival during the follow-up [HR 0.15 (95% CI 0.03–0.8), *P* = 0.03].

## Discussion

The present study reports an 11-year multicenter experience in the urgent surgical treatment of a significant number of PAAs (66) with a long follow-up period (52 ± 21 months). Few data are available in the literature about the long-term outcome of urgent PAAs treatment according to their clinical presentation (ALI or rupture).

As a matter of fact, popliteal artery aneurisms are preferably treated when asymptomatic with a diameter of ≥ 20 mm to prevent thrombotic complications or rupture and limb loss [6]. When ischemic symptoms due to aneurysm acute thrombosis or distal embolization (pain, coldness, sensory and/or motor loss) or symptoms related to PAA rupture (pain, calf swelling, adjacent structures compressive symptoms) occur, an urgent treatment is needed.

Endovascular repair using stent-graft deployment is increasing due to its low invasiveness. However, open surgery is still considered the gold standard, especially for symptomatic PAAs. It is reported that in case of acute limb ischemia the endovascular repair has lower patency (30-days 70.4% vs 93%, and 1-year 47.6% vs 86.8%, *P* = 0.001) and higher amputation rates (30-days 14.8% vs 3.7%, *P* = 0.022, and 1-year 17.4% vs 6.8%, *P* = 0.098) than open surgery [13]. Almost no data are available in the literature about the outcomes of endovascular repair in case of ruptured PAAs. According to the authors’ experience, it can be considered as an option in an emergency setting, but a surgical evacuation of the hematoma is often needed.

Rupture has been reported anecdotally and needs arterial ligation with or without bypass graft revascularization according to the clinical conditions of the patient. Most commonly symptomatic patients present with ALI. In those cases, it is of paramount importance to evaluate the degree of limb ischemia (mild, moderate, limb threatening, irreversible) to offer the best and timely treatment option [7].

In our series, preoperative intra-arterial thrombolysis was performed in 18 (33%) patients, and it was associated with higher long-term survival rate if compared to urgent bypass surgery alone (5-year survival 94 ± 6% vs. 62 ± 10%, *P* = 0.03). Thrombolysis may be used preoperatively in patients with mild to moderate ischemia (Rutherford I or IIa) to improve the tibio-pedal runoff, if not contraindicated [6, 7, 9]. Ravn et al. compared immediate surgical revascularization versus preoperative thrombolysis and delayed surgery in a population of 235 patients with ALI. This analysis showed that runoff improved in 87% of cases after thrombolysis and the amputation-rate was 27% in the first group and 7% in the second one (*P* < 0.0001) [10]. Similar results were reported by Dorigo et al. [11]. The authors underlined that successful preoperative intra-arterial thrombolysis provides better results than urgent surgery alone, without compromising the results of surgery if unsuccessful. Early limb salvage was 76% in the group undergoing urgent surgery alone and 86% in the preoperative thrombolysis and subsequent surgical revascularization group. Kropman et al. systematic review compared the same two groups of patients, but did not find a significant reduction in limb amputations in the group undergoing thrombolysis before surgery. However, the authors underlined that most of the included studies were based on retrospective data and they lacked important information (grade of ischemia, distal runoff, graft material) and long-term follow-up [12]. In case of limb threatening ischemia (Rutherford IIb [7]) a prompt revascularization is necessary and the effect of thrombolysis cannot be awaited; in these cases, intraoperative aspiration or mechanical thrombectomy can help restore the distal runoff, as was performed in the tibial arteries of 12 (22%) cases in our experience [6].

An interesting finding of our study is the independent association of number of patent tibial arteries (pTA) with long-term survival during follow-up. Moreover, the number of pTA was independently associated with limb salvage during follow-up. According to these data, in case of symptomatic PAA with ALI due to aneurysm thrombosis or distal embolization, obtaining as many pTA as possible by means of preoperative catheter-directed intra-arterial thrombolysis and/or intraoperative tibial arteries thrombectomy may provide better long-term survival and limb salvage rates.

In the present study only one (2%) death occurred in the first 30 days after surgery and overall 5-year survival was 70%. Ruptured PAAs were associated with lower long-term survival if compared to those with ALI at presentation (48% vs 79% at 5 years). According to our knowledge no data are available in literature about the long-term survival of urgently treated ruptured PAAs. It is worth noting that patients with ruptured PAA were older than those presenting with ischemic symptoms (82 vs 69 year-old, *P* = 0.01) in our series and this may be the reason for their shorter survival, as suggested by the lack of an independent association. This aspect was already underlined by Cervin et al. [13, 14], and it has a definite role on the long-term survival of this group of patients. On the other hand, the long-term survival of ALI patients is significantly longer, and our results are in line with those reported by Pulli et al. [15], with an estimated 60-month survival of 84.2%.

In the present experience the clinical presentation and the graft material of the bypass were not associated to limb loss during the follow-up period. Differently from other femoro-popliteal procedures, the saphenous vein did not show superior performance compared with synthetic material, probably due to the wider diameter of the arteries in the PAA pathology and the shorter length of the graft needed. As mentioned before, the only factor influencing the long-term limb salvage was the number of pTA.

The present study suffers of some limitations such as the retrospective analysis of data collected prospectively, the low number of patients due to the rarity of the pathology, and the data collection in two different vascular centers.

## Conclusions

Data about long-term outcomes of urgent PAA surgical treatment are limited; however, a prompt treatment ensures generally favorable outcomes and satisfactory long-term results. PAA rupture is not uncommon and accounts for nearly one fifth of cases requiring urgent treatment, often associated with low long-term survival rates. Patients experiencing ALI, but not facing limb threatening ischemia, can benefit from preoperative thrombolysis since their limb salvage and survival during follow-up are closely linked to the number of pTA.

## Data Availability

The datasets used and/or analyzed during the current study are available from the corresponding author on reasonable request.

## Abbreviations

PAAs: Popliteal artery aneurysms
ALI: Acute limb ischemia
CTA: Computed Tomography Angiography
GSV: Great saphenous vein
DUS: Duplex Ultrasound
ePTFE: Expanded polytetrafluoroethylene
HR: Hazard ratio
CI: Confidence interval
pTA: Patent tibial arteries
